# Postoperative delirium in orthopedic patients – identifying contributing factors and therapeutic strategies: A review

**DOI:** 10.1097/MD.0000000000046204

**Published:** 2026-05-12

**Authors:** Lian Huang, PengCheng Li, Jian Li

**Affiliations:** a Sports Medicine Center, West China Hospital, Sichuan University, Chengdu, China; b Department of Orthopedics and Orthopedic Research Institute, West China Hospital, Sichuan University, Chengdu, China; c West China School of Nursing, Sichuan University, Chengdu, China.

**Keywords:** orthopedic surgery, postoperative delirium, prevention, risk factors, treatment

## Abstract

Postoperative delirium (POD) is a common and serious neuropsychiatric complication following orthopedic surgeries, particularly in elderly patients. This integrative review aimed to provide a comprehensive synthesis of the current literature on the risk factors, pathophysiological mechanisms, prevention strategies, and management approaches for POD in orthopedic patients. The review identified a multitude of risk factors contributing to the development of POD, encompassing patient-related factors, preoperative factors, intraoperative factors, and postoperative factors The pathophysiology of POD is complex and involves an interplay of neurotransmitter imbalances, neuroinflammation, and oxidative stress. Prevention of POD requires a multimodal approach, incorporating both pharmacological interventions and non-pharmacological strategies. The management of POD necessitates a multidisciplinary approach, combining targeted pharmacotherapy with non-pharmacological interventions to control symptoms and promote recovery. Despite significant advancements in understanding POD, there remain gaps in knowledge that warrant further investigation. In conclusion, this review underscores the importance of implementing evidence-based, multicomponent interventions that integrate pharmacological and non-pharmacological strategies to reduce the incidence and severity of POD in orthopedic patients. Continued research efforts and dissemination of best practices are essential to mitigate the impact of this debilitating complication and improve patient outcomes.

## 1. Introduction

Postoperative delirium (POD) is a common clinical syndrome that significantly impacts patient outcomes following orthopedic surgery.^[1–4]^ Characterized by fluctuating levels of consciousness, inattention, and disorganized thinking, POD is particularly prevalent in elderly patients undergoing hip fracture surgery.^[5,6]^ This prevalence is largely attributed to factors such as advanced age, preoperative cognitive impairment, and the presence of multiple comorbidities. POD specifically refers to delirium occurring within the perioperative period following surgical procedures, distinct from ICU delirium or medical delirium in its temporal relationship to surgical trauma, anesthetic exposure, and specific risk factors related to orthopedic interventions. The incidence of POD in orthopedic surgery patients varies widely, reported between 4.5% and 41.2%,^[7–9]^ with an alarming statistic showing that 72.4% of those affected died within 5 years.^[10]^ The ramifications of POD are profound, encompassing immobilization, impaired functional recovery, extended hospital stays, increased mortality, and heightened healthcare costs.^[11]^ The high incidence and severe consequences of POD, especially in orthopedic surgeries, underscore the urgent need for a deeper understanding of its contributing factors and the development of effective therapeutic strategies.

Although the precise pathophysiological mechanisms underlying POD remain unclear, the condition has been linked to a variety of predisposing factors.^[12,13]^ These factors include older age, female gender, preexisting cognitive impairment, and complex comorbidities.^[14]^ Given the high incidence of POD, particularly in orthopedic surgery, and its association with increased morbidity and mortality, assessing the risk of delirium prior to surgery is crucial. However, existing studies exploring these risk factors are often limited by their scope and methodology.^[4,15]^ Many investigations have focused on a narrow range of potential risk factors or have been constrained by small sample sizes, limiting the generalizability of their findings.^[16,17]^ Furthermore, the results from individual studies have sometimes been inconsistent or even contradictory, adding to the uncertainty in this field. Consequently, there is still a significant degree of uncertainty regarding the predictive power of these individual risk factors for POD in the context of orthopedic surgery. This ambiguity underlines the necessity for a comprehensive and integrative review that can synthesize existing evidence and provide clearer insights into the risk factors and prevention strategies for POD in orthopedic patients.

In the realm of orthopedic surgery, few studies have comprehensively compared the types of surgeries, associated risk factors, and anesthesia and analgesia methods in relation to the development of POD. Recognizing this gap, our integrative review seeks to assess the incidence of POD following nonambulatory orthopedic surgeries. Our primary objectives are to evaluate how different surgical procedures influence the incidence of POD and to identify modifiable risk factors within this context. This approach aims to provide a more coherent understanding of POD in orthopedic patients, guiding more effective clinical decision-making and preventive strategies.

## 2. Materials and methods

This study was conducted using an integrative review approach, deemed the most effective for systematically synthesizing and analyzing both empirical and theoretical literature. This method was chosen to ensure that the findings would be directly relevant and applicable to clinical practices.

In aligning with the highest standards of research integrity, the article selection and literature review process adhered to the PRISMA (Preferred Reporting Items for Systematic Reviews and Meta-Analyses) guidelines. The methodological quality of the included studies was rigorously evaluated using the criteria set forth in the John Hopkins Nursing Evidence-Based Practice Model,^[18]^ ensuring a comprehensive and reliable analysis.

### 2.1. Search strategy

The literature search was meticulously executed, encompassing a decade-long period from January 2013 through November 2023. This timeframe was chosen to capture the most recent and relevant advancements in the field.

#### 2.1.1. Databases utilized

The search included key scientific databases known for their robust collection of medical and health-related literature, namely PubMed, Cochrane Library, Google Scholar, and CINAHL.

#### 2.1.2. Search terms

The search strategy was built around specific terms central to our study’s theme. These included “Postoperative Delirium” (MeSH term: D003693), “Orthopedic Surgical Procedures” (MeSH term: D019637), and “Delirium, Emergence” (MeSH term: D000071257). Additional related terms incorporated were “Anaesthesia delirium,” “Surgery delirium,” and “Orthopedic Patients.”

#### 2.1.3. Boolean logic

Boolean operators were employed to broaden or narrow the search as needed. “OR” was used to link similar concepts, while “AND” combined different aspects of the topic, enhancing the search’s specificity and relevance.

#### 2.1.4. Filters and limits

The search was refined using specific filters, including full-text availability, human studies, articles in English, and publications from January 2013 to November 2023. Studies involving pediatric subjects were excluded to maintain the focus on adult orthopedic patients.

#### 2.1.5. Example of a PubMed search

A representative search in PubMed might include “Postoperative Delirium” AND “Orthopedic Surgery” with the specified time filter, full-text availability, human studies, and English language articles.

#### 2.1.6. Inclusion and exclusion criteria

The review included studies pertinent to POD in adult orthopedic patients, with a focus on identifying contributing factors and therapeutic strategies. Exclusion criteria encompassed non-English articles, pediatric-focused research, and studies outside the designated time range.

This search strategy was designed to be comprehensive, ensuring a thorough exploration of the literature from the past decade, thereby facilitating a detailed understanding of POD in orthopedic patients and aiding in the identification of critical contributing factors and effective therapeutic interventions.

### 2.2. Selection criteria

#### 2.2.1. Time frame

The literature search for the integrative review was focused on articles published from 2013 to 2023. This time frame was strategically chosen to encompass recent advancements in the field. While acknowledging previous reviews spanning the last 18 years, it was observed that these did not provide a comprehensive summary of the subject, and numerous high-quality studies published in recent years were not included.

#### 2.2.2. Language

Articles published in English were primarily considered, ensuring wide accessibility and relevance to a broad scientific audience.

#### 2.2.3. Study types

The review included a diverse range of study designs to capture a comprehensive view of the topic. This encompassed randomized clinical trials, observational studies, systematic reviews, integrative reviews, and bibliographic reviews. Emphasis was placed on studies offering empirical evidence and theoretical insights.

#### 2.2.4. Content focus

The articles selected were specifically related to POD in orthopedic patients. Studies that extensively discussed contributing factors, therapeutic strategies, both pharmacological and non-pharmacological interventions, and diagnostic methods were prioritized.

#### 2.2.5. Population

This review targeted adult patients of all ages undergoing orthopedic surgery, recognizing that POD affects a broad age spectrum. While older adults, particularly those over 65, are at higher risk, the inclusion of younger adults addressed the variability of delirium incidence across different age groups.

#### 2.2.6. Exclusion criteria

Articles that did not directly address the topic of POD in orthopedic patients, were outside the specified time frame, or were not in English were excluded. Additionally, studies with a primary focus on pediatric populations were omitted.

### 2.3. Article selection and data extraction

To ensure a rigorous and unbiased selection process, the initial literature search was conducted independently by two reviewers using the predefined search strategy across the specified databases. After removing duplicate records, the first round of screening was performed by independently evaluating the titles of the retrieved articles.

Subsequently, a second screening was carried out, which involved carefully examining the abstracts and keywords of the articles shortlisted by both reviewers. This step culminated in the creation of a refined list of articles for further consideration. Any disagreements between the reviewers were resolved through discussion and consensus.

Upon procuring the full-text versions of the selected articles, a third and final round of screening was conducted to determine the ultimate set of studies to be included in this systematic review.

Lastly, after finalizing the articles for inclusion, relevant data were extracted from each study, including publication date, article title, study design, population characteristics, intervention type, outcome variables, results, influencing factors, pharmacological interventions, medications that decreased the incidence of POD, non-pharmacological interventions, and diagnostic tests or scales employed. This process was undertaken to facilitate the synthesis and management of the collected information.

Due to the substantial heterogeneity among the included studies, particularly in terms of interventions and assessment methods, conducting a meta-analysis was deemed infeasible. The methodological diversity (RCTs vs observational studies) was addressed by stratifying findings by study design and weighting conclusions based on evidence quality, with randomized controlled trial findings given priority for intervention recommendations while observational data informed risk factor identification. Alternative quantitative synthesis methods (subgroup analysis, narrative synthesis) were considered but deemed inappropriate due to the diversity in outcome measures, patient populations (age ranges, surgical types), and inconsistent reporting of effect sizes across studies.

### 2.4. Characterization of articles

**Figure 1.**
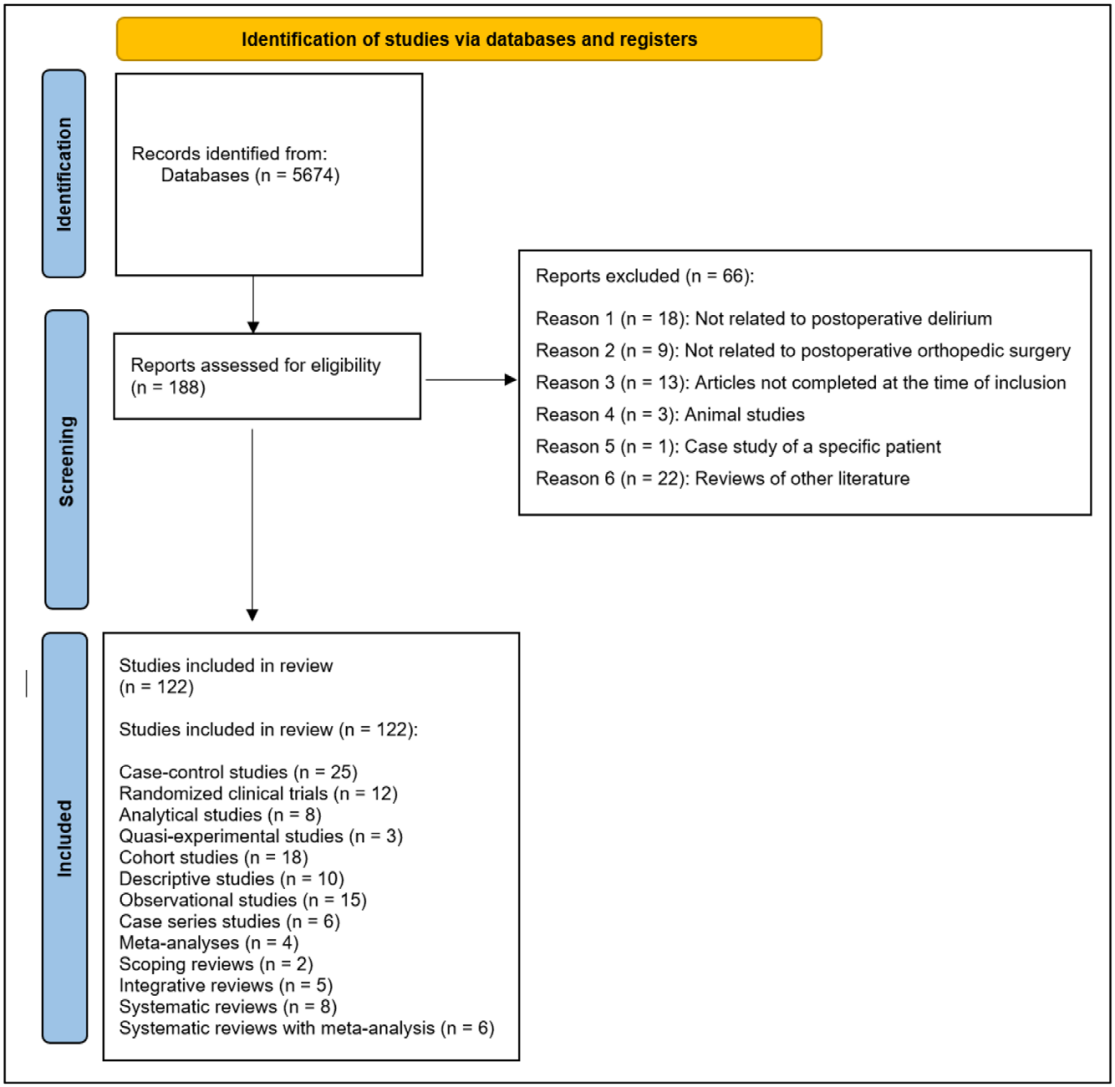
PRISMA flow chart showing the results of the search completed. PRISMA = Preferred Reporting Items for Systematic Reviews and Meta-Analyses.

Figure 1 illustrates the PRISMA flow diagram of the results obtained from the search process. The initial search yielded a total of 5674 results. A total of 188 articles met the inclusion criteria, and their full-text versions were obtained. After a thorough analysis, 66 articles were excluded for the following reasons: 18 were not related to POD, 9 were not related to postoperative orthopedic surgery, 13 articles were conference abstracts or protocols without full published results available at the time of inclusion, 3 were animal studies, 1 was a case study of a specific patient, and 22 were reviews of other literature (Fig. 1).

The final selection included 122 articles, distributed as follows: 25 case-control studies, 12 randomized clinical trials, 8 analytical studies, 3 quasi-experimental studies, 18 cohort studies, 10 descriptive studies, 15 observational studies, 6 case series studies, 4 meta-analyses, 2 scoping reviews, 5 integrative reviews, 8 systematic reviews, and 6 systematic reviews with meta-analysis.

### 2.5. Assessment of methodological quality

To appraise the methodological quality of the included studies, we employed the widely accepted John Hopkins Nursing Evidence-Based Practice Model.^[19]^ This model provides a framework for assigning a level of evidence (I, II, III, IV, or V) and a quality rating (A for high-quality, B for good quality, or C for low quality or major flaws) to each study based on its design and the robustness of the evidence presented.

The John Hopkins Nursing Evidence-Based Practice Model offers a systematic approach to evaluating the strength and quality of evidence, allowing for a more objective assessment of the studies included in this systematic review. By applying this model, we were able to critically examine the methodological rigor of each study and determine its contribution to the overall body of evidence on POD in orthopedic patients. Evidence quality directly influenced our recommendations: level I and II evidence (systematic reviews, RCTs) formed the basis for prevention and treatment recommendations, while level III and IV evidence (observational studies) primarily informed risk factor identification and pathophysiology discussions. High-quality evidence (grade A) was prioritized in clinical recommendation formulation.

## 3. Results

### 3.1. Risk factors for postoperative delirium in orthopedic patients

#### 3.1.1. Patient-related factors

Advanced age is a well-established risk factor for POD in orthopedic patients. Several studies have consistently demonstrated that the incidence of POD increases with age, with patients over 65 years being at a significantly higher risk.^[1,20]^ The aging brain is more susceptible to the effects of anesthesia, surgical stress, and inflammation, which may contribute to the development of POD.^[21]^ Additionally, age-related changes in neurotransmitter systems, particularly cholinergic pathways, have been implicated in the pathogenesis of delirium.^[22]^

Preexisting cognitive impairment, such as dementia or mild cognitive impairment, is another important patient-related risk factor for POD.^[23]^ Patients with preexisting cognitive deficits have a reduced cognitive reserve and are more vulnerable to the insults associated with surgery and anesthesia.^[24]^ Moreover, these patients may have difficulty in understanding and adhering to postoperative instructions, which can further increase their risk of developing POD.^[25]^

Comorbidities, including depression, diabetes, and cardiovascular disease, have also been associated with an increased risk of POD in orthopedic patients.^[4,26]^ Depression has been linked to alterations in neurotransmitter systems and immune function, which may predispose patients to POD.^[27]^ Diabetes can lead to microvascular changes and impaired glucose metabolism in the brain, potentially contributing to the development of delirium.^[28]^ Cardiovascular disease, particularly coronary artery disease and heart failure, may cause cerebral hypoperfusion and increase the risk of POD.^[29]^

Other patient-related factors that have been associated with POD include functional dependence, sensory impairments (visual and hearing), and a history of alcohol abuse.^[30,31]^ Patients with functional limitations may have difficulty in mobilizing and participating in postoperative care, which can increase their risk of POD.^[32]^ Sensory impairments can hinder the patient’s ability to orient themselves and interact with their environment, leading to confusion and delirium.^[33]^ Alcohol abuse has been linked to neurotoxicity, nutritional deficiencies, and withdrawal syndromes, all of which can contribute to the development of POD.^[34]^

#### 3.1.2. Preoperative factors

Inadequate pain control before surgery is a significant risk factor for POD in orthopedic patients.^[35]^ Preoperative pain can lead to increased stress response, sleep disturbances, and anxiety, all of which have been associated with the development of POD.^[36]^ Moreover, poorly controlled pain may necessitate the use of higher doses of opioids, which can further increase the risk of delirium.^[37]^

Certain medications, when used preoperatively, have been linked to a higher incidence of POD. Benzodiazepines, especially long-acting agents, have been associated with an increased risk of POD, possibly due to their effects on cognitive function and sedation.^[38]^ Anticholinergic medications, such as antihistamines and tricyclic antidepressants, can also increase the risk of POD by interfering with neurotransmitter balance in the brain.^[39]^ The use of opioids, particularly in high doses, has been associated with an increased risk of POD, likely due to their effects on cognitive function and respiratory depression.^[40]^

Poor nutritional status, as indicated by low serum albumin levels or malnutrition, has been identified as a risk factor for POD.^[41]^ Malnutrition can lead to impaired immune function, delayed wound healing, and reduced cognitive reserve, all of which can contribute to the development of POD.^[42]^ Preoperative anemia has also been associated with an increased risk of POD, possibly due to reduced oxygen delivery to the brain.^[43]^

Other preoperative factors that have been linked to POD include dehydration, electrolyte imbalances, and urinary tract infections.^[11,44]^ Dehydration can cause cognitive dysfunction and increase the risk of postoperative complications, including POD. Electrolyte imbalances, particularly hyponatremia and hypocalcemia, have been associated with an increased risk of POD, possibly due to their effects on neurotransmitter function.^[45]^ Urinary tract infections can lead to systemic inflammation and have been linked to an increased risk of POD, especially in elderly patients.^[46]^

#### 3.1.3. Intraoperative factors

The type of anesthesia used during orthopedic surgery may influence the risk of POD. Some studies suggest that general anesthesia is associated with a higher incidence of POD compared to regional anesthesia.^[47]^ This increased risk may be attributed to the systemic effects of general anesthetics on the brain, including alterations in neurotransmitter levels and cerebral blood flow.^[48]^ However, the evidence regarding the impact of anesthesia type on POD is not conclusive, with some studies reporting no significant difference between general and regional anesthesia.^[49]^

The depth of anesthesia during surgery has also been investigated as a potential risk factor for POD. Intraoperative burst suppression, a pattern on electroencephalography (EEG) indicative of deep anesthesia, has been associated with an increased risk of POD.^[50]^ This finding suggests that excessive anesthetic depth may contribute to the development of POD, possibly through its effects on brain function and recovery.^[51]^

Prolonged surgery duration has been identified as another risk factor for POD in orthopedic patients.^[52]^ Longer procedures may increase the exposure to anesthetic agents, lead to greater blood loss, and cause more extensive surgical trauma, all of which can contribute to the development of POD.^[53]^ Additionally, prolonged surgery may be associated with increased postoperative pain and inflammation, which have been linked to POD.^[20]^

Intraoperative blood loss and the need for blood transfusions have also been associated with an increased risk of POD in orthopedic surgery.^[54]^ Substantial blood loss can lead to anemia, reduced oxygen delivery to the brain, and increased risk of complications, such as hypotension and organ dysfunction.^[55]^ Blood transfusions, while necessary to correct anemia, have been independently linked to POD, possibly due to the immunomodulatory effects of transfused blood products.^[56]^

Intraoperative hypotension, defined as a significant decrease in blood pressure from baseline, has been identified as a risk factor for POD.^[57]^ Hypotension can lead to decreased cerebral perfusion and oxygenation, which may contribute to the development of POD.^[58]^ Maintaining adequate blood pressure and cerebral perfusion during surgery is crucial for reducing the risk of POD.^[59]^

Other intraoperative factors that have been associated with POD include hypothermia, hyperglycemia, and the use of certain medications, such as anticholinergics and benzodiazepines.^[56,60]^ Hypothermia can lead to shivering, increased oxygen consumption, and altered drug metabolism, all of which may contribute to POD.^[61]^ Hyperglycemia has been linked to increased inflammation and oxidative stress, which may adversely affect brain function and increase the risk of POD.^[62]^ The use of medications with anticholinergic properties or sedative effects during surgery may also increase the risk of POD, particularly in older patients.^[22]^

#### 3.1.4. Postoperative factors

Inadequate postoperative pain control is a significant risk factor for the development of POD in orthopedic patients.^[36]^ Uncontrolled pain can lead to increased stress, sleep disturbances, and impaired mobility, all of which have been associated with an increased risk of POD.^[63]^ Moreover, the use of opioids for postoperative pain management may further contribute to the development of POD, particularly in elderly patients who are more susceptible to the adverse effects of these medications.^[40]^

Sleep disturbances, including sleep fragmentation and reduced sleep quality, are common in the postoperative period and have been independently associated with an increased risk of POD.^[64]^ Disrupted sleep can lead to cognitive dysfunction, impaired memory consolidation, and altered neurotransmitter levels, which may contribute to the development of POD.^[65]^ Implementing strategies to promote sleep hygiene and minimize sleep disruptions in the postoperative setting may help reduce the risk of POD.^[66]^

Postoperative immobility and reduced physical activity have been linked to an increased risk of POD in orthopedic patients.^[67]^ Prolonged bed rest and lack of early mobilization can lead to muscle weakness, functional decline, and increased risk of complications, such as deep vein thrombosis and pneumonia.^[68]^ Early mobilization and physical therapy interventions have been shown to reduce the incidence and duration of POD, possibly by promoting functional recovery and reducing the risk of complications.^[69]^

Postoperative infections, including surgical site infections and urinary tract infections, have been associated with an increased risk of POD in orthopedic patients.^[60]^ Infections can trigger systemic inflammation, which may adversely affect brain function and contribute to the development of POD.^[21]^ Prompt diagnosis and treatment of postoperative infections, as well as the implementation of infection prevention measures, are essential for reducing the risk of POD.^[70]^

Electrolyte imbalances, particularly hyponatremia and hypocalcemia, are common in the postoperative period and have been linked to an increased risk of POD.^[71]^ These imbalances can affect neurotransmitter function, leading to cognitive dysfunction and delirium.^[44]^ Close monitoring and correction of electrolyte abnormalities in the postoperative period may help reduce the risk of POD.^[72]^

Postoperative anemia, often resulting from surgical blood loss and hemodilution, has been identified as a risk factor for POD in orthopedic patients.^[73]^ Anemia can lead to reduced oxygen delivery to the brain, which may contribute to the development of POD.^[74]^ Strategies to minimize blood loss during surgery, as well as the judicious use of blood transfusions in the postoperative period, may help reduce the risk of POD.^[75]^

Other postoperative factors that have been associated with an increased risk of POD include dehydration, malnutrition, and the use of certain medications, such as anticholinergics and benzodiazepines.^[42]^ Maintaining adequate hydration and nutrition in the postoperative period, as well as carefully selecting and monitoring medications, may help reduce the risk of POD^[76]^ (Table 1).

**Table 1:** Risk factors for postoperative delirium in orthopedic patients.

Category	Risk factors	Reported OR range	Evidence quality	References
Patient-related factors	Advanced age	1.5–3.2	High	[1,20]
	Preexisting cognitive impairment	2.1–4.8	High	[23–25]
	Comorbidities (depression, diabetes, cardiovascular disease)	1.4–2.6	Moderate	[4,26–29]
	Functional dependence	1.6–2.9	Moderate	[30,32]
	Sensory impairments (visual and hearing)	1.3–2.1	Low	[30,33]
	History of alcohol abuse	1.8–3.4	Low	[30,34]
Preoperative factors	Inadequate pain control	1.9–3.1	Moderate	[35–37]
	Medication use (benzodiazepines, anticholinergics, opioids)	2.3–4.1	High	[38–40]
	Poor nutritional status	1.7–2.8	Moderate	[41,42]
	Preoperative anemia	1.4–2.2	Low	[43]
	Dehydration	1.5–2.4	Low	[11,44]
	Electrolyte imbalances	1.6–2.7	Moderate	[44,45]
	Urinary tract infections	1.8–2.9	Low	[46]
Intraoperative factors	General anesthesia (controversial)	1.2–1.8	Low	[47–49]
	Deep anesthesia (intraoperative burst suppression)	1.7–2.8	Moderate	[50,51]
	Prolonged surgery duration	1.5–2.6	Moderate	[52,53]
	Increased intraoperative blood loss and blood transfusions	1.8–3.4	Moderate	[54–56]
	Intraoperative hypotension	1.4–2.3	Moderate	[57–59]
	Hypothermia	1.3–2.1	Low	[60,61]
	Hyperglycemia	1.5–2.4	Low	[62]
	Use of anticholinergics and benzodiazepines	2.1–3.8	Moderate	[22]
Postoperative factors	Inadequate pain control	2.1–3.7	High	[36,63]
	Sleep disturbances and reduced sleep quality	1.9–2.8	Moderate	[64–66]
	Immobility and reduced physical activity	2.4–4.2	Moderate	[67–69]
	Postoperative infections (surgical site infections, urinary tract infections)	1.7–2.9	Moderate	[60,70]
	- Electrolyte imbalances (hyponatremia, hypocalcemia)	1.6–2.5	Moderate	[71,72]
	Postoperative anemia	1.5–2.3	Low	[73–75]
	Dehydration	1.4–2.2	Low	[76]
	Malnutrition	1.8–2.7	Low	[42]
	Use of anticholinergics and benzodiazepines	2.2–3.9	Moderate	[76]

Due to heterogeneity in outcome reporting across studies, effect sizes represent typical ranges reported in multiple studies. Evidence quality: high (consistent across RCTs/large cohorts), moderate (some inconsistency), low (limited/conflicting data).

OR = odds ratio, RCT = randomized controlled trial.

### 3.2. Pathophysiology of postoperative delirium in orthopedic patients

While the molecular mechanisms remain incompletely understood, current evidence from orthopedic surgery contexts suggests the following pathways are most clinically relevant.

#### 3.2.1. Neurotransmitter imbalances

Neurotransmitter imbalances play a crucial role in the pathophysiology of POD in orthopedic patients. The disruption of normal neurotransmitter signaling, particularly involving acetylcholine, dopamine, and serotonin, has been implicated in the development of POD.^[21]^

Acetylcholine is a key neurotransmitter involved in cognitive function, attention, and memory. The cholinergic deficiency hypothesis suggests that a decrease in acetylcholine levels or impaired cholinergic neurotransmission contributes to the development of delirium.^[22]^ In orthopedic surgery, the use of anticholinergic medications, such as opioids and benzodiazepines, for pain management and sedation may exacerbate this cholinergic deficiency, increasing the risk of POD.^[39]^ Additionally, the stress response associated with surgery and anesthesia can lead to increased cortisol levels, which have been shown to inhibit acetylcholine release, further contributing to the cholinergic deficit.^[77]^

Dopamine, another essential neurotransmitter, is involved in cognitive processes, motor function, and reward-seeking behavior. An imbalance in dopaminergic neurotransmission, characterized by either an excess or deficiency of dopamine, has been linked to the development of POD.^[78]^ In the context of orthopedic surgery, the use of dopamine-modulating medications, such as antipsychotics and antiparkinsonian drugs, may disrupt the delicate balance of dopaminergic signaling.^[79]^ Moreover, the inflammatory response triggered by surgical trauma and tissue damage can lead to alterations in dopamine metabolism, further contributing to the dopaminergic imbalance.^[80]^

Serotonin, a monoamine neurotransmitter, plays a role in mood regulation, sleep-wake cycles, and cognitive function. Disturbances in serotonergic neurotransmission have been associated with the development of POD.^[81]^ In orthopedic patients, the use of serotonergic medications, such as selective serotonin reuptake inhibitors and opioids, may contribute to serotonergic imbalances.^[40]^ Additionally, the inflammatory response and oxidative stress associated with orthopedic surgery can lead to alterations in serotonin metabolism, further disrupting serotonergic signaling.^[82]^

The complex interplay between these neurotransmitter systems and their imbalances in the context of orthopedic surgery may contribute to the development of POD. For example, the cholinergic deficiency may lead to a relative excess of dopaminergic signaling, resulting in the characteristic symptoms of POD, such as inattention, disorganized thinking, and perceptual disturbances.^[83]^ Furthermore, the serotonergic imbalance may exacerbate the cholinergic deficiency and dopaminergic excess, creating a vicious cycle that perpetuates the delirious state.^[84]^

Understanding the role of neurotransmitter imbalances in the pathophysiology of POD in orthopedic patients is crucial for developing targeted interventions and preventive strategies. For instance, minimizing the use of medications that disrupt cholinergic, dopaminergic, and serotonergic signaling, as well as implementing non-pharmacological interventions that promote neurotransmitter balance, such as early mobilization and cognitive stimulation, may help reduce the risk of POD in this vulnerable population.^[1]^

#### 3.2.2. Neuroinflammation

Neuroinflammation plays a pivotal role in the pathophysiology of POD in orthopedic patients. The inflammatory response triggered by surgical trauma, anesthesia, and postoperative pain can lead to a cascade of events that ultimately affect brain function and contribute to the development of POD.^[21]^

In the context of orthopedic surgery, tissue damage and the release of damage-associated molecular patterns (DAMPs) initiate a local inflammatory response. This response is characterized by the activation of resident immune cells, such as macrophages and mast cells, and the release of pro-inflammatory cytokines, including interleukin-1β (IL-1β), interleukin-6, and tumor necrosis factor-α.^[85]^ These cytokines can cross the blood–brain barrier and activate microglial cells, the resident immune cells of the central nervous system.^[86]^

Activated microglia undergo morphological changes and release additional pro-inflammatory cytokines and chemokines, perpetuating the inflammatory response within the brain.^[87]^ This neuroinflammatory cascade can lead to neurotransmitter imbalances, particularly affecting the cholinergic and dopaminergic systems, which are crucial for cognitive function and attention.^[80]^ Moreover, the release of reactive oxygen species (ROS) and nitric oxide by activated microglia can cause oxidative stress and further exacerbate neuronal dysfunction.^[88]^

The aging brain is particularly susceptible to the detrimental effects of neuroinflammation. In elderly orthopedic patients, age-related changes in the immune system, such as immunosenescence and inflammaging, may exacerbate the inflammatory response to surgical insults.^[89]^ Additionally, preexisting neuroinflammatory conditions, such as Alzheimer’s disease and other neurodegenerative disorders, can increase the vulnerability of the brain to the effects of surgery-induced inflammation.^[90]^

The impact of neuroinflammation on the blood–brain barrier (BBB) integrity is another critical aspect of POD pathophysiology. The BBB is a highly selective semipermeable border that separates the circulating blood from the brain and extracellular fluid in the central nervous system, maintaining a stable microenvironment for proper neuronal function.^[91]^ Neuroinflammation can lead to increased BBB permeability, allowing the infiltration of peripheral immune cells and inflammatory mediators into the brain parenchyma.^[92]^ This disruption of BBB integrity can further exacerbate neuroinflammation and contribute to the development of POD.

The neuroinflammatory response may also be modulated by genetic factors, such as polymorphisms in genes encoding pro-inflammatory cytokines and their receptors. For example, the IL-1β gene polymorphism has been associated with an increased risk of POD in elderly patients undergoing hip fracture surgery.^[93]^ These genetic variations may explain, in part, the individual differences in susceptibility to POD among orthopedic patients.

Understanding the complex role of neuroinflammation in the pathophysiology of POD in orthopedic patients has important clinical implications. Strategies aimed at modulating the inflammatory response, such as the use of anti-inflammatory agents (e.g., nonsteroidal anti-inflammatory drugs, statins, and corticosteroids), may help reduce the risk of POD.^[94]^ Additionally, non-pharmacological interventions that promote the resolution of inflammation, such as early mobilization and physiotherapy, may contribute to the prevention and management of POD in this vulnerable population.^[1]^

In conclusion, neuroinflammation is a key pathophysiological mechanism underlying the development of POD in orthopedic patients. The complex interplay between surgical trauma, anesthesia, pain, and the aging brain creates a pro-inflammatory milieu that can lead to neurotransmitter imbalances, oxidative stress, and BBB disruption. Understanding these processes is crucial for developing targeted interventions and preventive strategies to reduce the burden of POD in orthopedic patients.

#### 3.2.3. Oxidative stress

Oxidative stress is another critical pathophysiological mechanism involved in the development of POD in orthopedic patients. It occurs when there is an imbalance between the production of ROS and the body’s ability to neutralize them through antioxidant defenses.^[95]^ In the context of orthopedic surgery, oxidative stress can be triggered by various factors, including surgical trauma, ischemia-reperfusion injury, anesthesia, and the inflammatory response.^[96]^

During orthopedic surgery, tissue damage and ischemia-reperfusion injury can lead to the generation of ROS, such as superoxide anion (O_2_^•−^), hydrogen peroxide (H_2_O_2_), and hydroxyl radical (•OH).^[97]^ These highly reactive molecules can cause lipid peroxidation, protein oxidation, and DNA damage, leading to cellular dysfunction and apoptosis.^[98]^ The brain is particularly vulnerable to oxidative stress due to its high metabolic rate, rich lipid content, and relatively low antioxidant capacity.^[99]^

The inflammatory response triggered by surgical trauma and tissue damage can also contribute to oxidative stress. Activated immune cells, such as neutrophils and macrophages, release ROS as part of their antimicrobial defense mechanisms.^[100]^ However, excessive production of ROS can lead to collateral damage to the surrounding tissues, including the brain.^[101]^ Moreover, pro-inflammatory cytokines, such as IL-1β and TNF-α, can induce the expression of ROS-generating enzymes, such as NADPH oxidase and inducible nitric oxide synthase, further exacerbating oxidative stress.^[102]^

Anesthesia, particularly the use of volatile anesthetics, has been associated with increased oxidative stress.^[103]^ These agents can impair mitochondrial function, leading to increased ROS production and decreased ATP synthesis.^[104]^ Additionally, some anesthetics, such as sevoflurane, have been shown to induce neuroapoptosis and cognitive dysfunction in animal models, possibly through oxidative stress-mediated mechanisms.^[105]^

The aging brain is more susceptible to the deleterious effects of oxidative stress. With advancing age, there is a decline in the efficiency of antioxidant defense systems, such as glutathione, superoxide dismutase, and catalase.^[106]^ This age-related reduction in antioxidant capacity, coupled with increased ROS production, can render the elderly brain more vulnerable to oxidative damage and cognitive dysfunction.^[107]^

Oxidative stress can also contribute to the disruption of the blood–brain barrier (BBB) integrity, which is a hallmark of neuroinflammation and a potential mechanism underlying POD.^[108]^ ROS can cause endothelial dysfunction and increase BBB permeability, allowing the infiltration of peripheral immune cells and inflammatory mediators into the brain.^[109]^ This breakdown of the BBB can further exacerbate oxidative stress and neuroinflammation, creating a vicious cycle that promotes cognitive dysfunction.^[110]^

The role of oxidative stress in the pathophysiology of POD has important implications for the development of preventive and therapeutic strategies. Antioxidants, such as vitamin C, vitamin E, and *N*-acetylcysteine (NAC), have been shown to attenuate oxidative stress and cognitive dysfunction in animal models of surgery-induced neuroinflammation.^[111]^ Additionally, the use of anesthetic agents with less potential for inducing oxidative stress, such as propofol, may help reduce the risk of POD in orthopedic patients.^[112]^

Non-pharmacological interventions that promote the resolution of oxidative stress, such as early mobilization and physical exercise, may also contribute to the prevention and management of POD.^[113]^ Exercise has been shown to upregulate antioxidant enzymes, such as superoxide dismutase and glutathione peroxidase, and to reduce oxidative stress markers in the brain.

In conclusion, oxidative stress is a key pathophysiological mechanism underlying the development of POD in orthopedic patients. The complex interplay between surgical trauma, ischemia-reperfusion injury, inflammation, and anesthesia creates an environment conducive to the generation of ROS and the impairment of antioxidant defenses. The resulting oxidative damage can lead to neuronal dysfunction, BBB disruption, and cognitive impairment. Understanding the role of oxidative stress in POD is crucial for developing targeted interventions and preventive strategies to reduce the burden of this complication in orthopedic patients.

### 3.3. Prevention strategies for postoperative delirium in orthopedic patients

#### 3.3.1. Pharmacological interventions

Pharmacological interventions play a crucial role in the prevention of POD in orthopedic patients. Several classes of drugs have been investigated for their potential to reduce the incidence and severity of POD, including antipsychotics, cholinesterase inhibitors, melatonin, and statins.^[20]^

Antipsychotics, particularly haloperidol and atypical antipsychotics such as risperidone and olanzapine, have been extensively studied for the prevention of POD. These medications are thought to work by modulating the imbalance in neurotransmitter systems, particularly dopamine and serotonin, which are implicated in the pathophysiology of delirium.^[21]^ A meta-analysis of randomized controlled trials found that prophylactic use of antipsychotics significantly reduced the incidence of POD in elderly patients undergoing surgery.^[114]^ However, the use of antipsychotics may be associated with adverse effects, such as extrapyramidal symptoms and prolonged QTc interval, warranting careful patient selection and monitoring.^[115]^

Cholinesterase inhibitors, such as donepezil and rivastigmine, have been investigated for their potential to prevent POD by enhancing cholinergic neurotransmission. The cholinergic deficiency hypothesis suggests that a decrease in acetylcholine levels contributes to the development of delirium.^[22]^ A systematic review and meta-analysis found that prophylactic use of cholinesterase inhibitors may reduce the incidence of POD in elderly patients undergoing elective surgery, although the quality of evidence was low.^[116]^ Further high-quality studies are needed to confirm the efficacy and safety of cholinesterase inhibitors in the prevention of POD.

Melatonin, a hormone involved in the regulation of sleep-wake cycles, has been investigated for its potential to prevent POD. Sleep disturbances and circadian rhythm disruptions are common in the postoperative period and have been associated with an increased risk of POD.^[64]^ Melatonin has been shown to have antioxidant, anti-inflammatory, and neuroprotective properties, which may contribute to its potential benefits in the prevention of POD.^[117]^ A systematic review and meta-analysis found that perioperative melatonin administration significantly reduced the incidence of POD in elderly patients undergoing surgery.^[118]^ However, The optimal dosing protocols and patient selection criteria for melatonin in orthopedic surgery require further investigation.

Statins, a class of lipid-lowering medications, have been investigated for their potential to prevent POD due to their pleiotropic effects, including anti-inflammatory and neuroprotective properties.^[119]^ Statins have been shown to reduce oxidative stress, attenuate neuroinflammation, and improve endothelial function, which may contribute to their potential benefits in the prevention of POD.^[120]^ A meta-analysis of observational studies found that preoperative statin use was associated with a reduced risk of POD in patients undergoing cardiac surgery.^[121]^ However, randomized controlled trials are needed to confirm the efficacy of statins in the prevention of POD in orthopedic patients. However, the evidence for statins remains limited to observational studies with potential confounding factors, and randomized trials in orthopedic populations are lacking.

Other pharmacological agents that have been investigated for the prevention of POD include dexmedetomidine, gabapentin, and ketamine. Dexmedetomidine, a selective α-2 adrenergic receptor agonist, has been shown to reduce the incidence of POD in patients undergoing cardiac and noncardiac surgery.^[122]^ Gabapentin, an anticonvulsant and analgesic, has been associated with a reduced risk of POD in patients undergoing spine surgery.^[123]^ Ketamine, an *N*-methyl-d-aspartate receptor antagonist, has been shown to have neuroprotective properties and may reduce the incidence of POD in patients undergoing cardiac surgery.^[124]^ However, further studies are needed to confirm the efficacy and safety of these agents in the prevention of POD in orthopedic patients.

In conclusion, pharmacological interventions have the potential to prevent POD in orthopedic patients by targeting the underlying pathophysiological mechanisms, such as neurotransmitter imbalances, neuroinflammation, and oxidative stress. Antipsychotics, cholinesterase inhibitors, melatonin, and statins have shown promising results in reducing the incidence of POD, although further high-quality studies are needed to confirm their efficacy and safety. The optimal pharmacological strategy for the prevention of POD in orthopedic patients may involve a combination of agents targeting multiple pathophysiological pathways, along with careful patient selection and monitoring for adverse effects.

#### 3.3.2. Non-pharmacological approaches

Non-pharmacological interventions play a crucial role in the prevention of POD in orthopedic patients. These approaches focus on modifying environmental factors, optimizing patient care, and promoting cognitive and physical function.^[1]^ Non-pharmacological strategies are particularly important as they can be implemented in conjunction with pharmacological interventions and have the potential to reduce the need for medications that may have adverse effects.^[115]^

One of the key non-pharmacological approaches for preventing POD is early mobilization. Prolonged immobilization and bed rest have been associated with an increased risk of POD, likely due to their effects on cognitive and physical function.^[125]^ Early mobilization, which involves getting patients out of bed and engaging them in physical activity as soon as possible after surgery, has been shown to reduce the incidence and duration of POD.^[126]^ A systematic review and meta-analysis found that early mobilization significantly reduced the incidence of POD in elderly patients undergoing hip fracture surgery.^[127]^ Implementing early mobilization protocols and encouraging patients to engage in physical activity can help prevent POD in orthopedic patients. Practical implementation typically involves: mobilization within 24 to 48 hours post-surgery when medically stable, progressive activity progression from sitting to standing to walking, and multidisciplinary coordination between nursing, physiotherapy, and surgical teams.

Cognitive stimulation and orientation interventions are another important non-pharmacological strategy for preventing POD. These interventions aim to keep patients mentally engaged and oriented to their surroundings, which can help prevent cognitive decline and confusion.^[114]^ Examples of cognitive stimulation interventions include providing patients with clocks and calendars, engaging them in conversations about current events, and encouraging them to participate in mentally stimulating activities such as puzzles and games.^[128]^ Orientation interventions involve regularly reorienting patients to their surroundings, reminding them of the date and time, and providing them with familiar objects from home.^[129]^ A systematic review and meta-analysis found that cognitive stimulation and orientation interventions significantly reduced the incidence of POD in elderly patients undergoing surgery.^[130]^

Sensory enhancement interventions, such as providing patients with hearing aids and eyeglasses, can also help prevent POD. Sensory impairments, particularly visual and hearing impairments, have been associated with an increased risk of POD.^[26]^ By ensuring that patients have access to their sensory aids and that their sensory needs are met, healthcare providers can help reduce the risk of POD.^[131]^ Additionally, creating a sensory-friendly environment, such as minimizing noise and providing adequate lighting, can help prevent sensory overstimulation and reduce the risk of POD.^[132]^

Sleep promotion interventions are another important non-pharmacological strategy for preventing POD. Sleep disturbances and circadian rhythm disruptions are common in the postoperative period and have been associated with an increased risk of POD.^[65]^ Non-pharmacological interventions to promote sleep include establishing a regular sleep-wake schedule, minimizing nighttime disruptions, and creating a sleep-conducive environment (e.g., reducing noise and light levels).^[133]^ A systematic review and meta-analysis found that non-pharmacological sleep promotion interventions significantly reduced the incidence of POD in hospitalized patients.^[134]^

Nutritional interventions, such as ensuring adequate hydration and providing high-protein supplements, may also help prevent POD. Malnutrition and dehydration have been associated with an increased risk of POD, likely due to their effects on cognitive and physical function.^[42]^ By monitoring patients’ nutritional status and providing them with appropriate nutrition and hydration, healthcare providers can help reduce the risk of POD.^[135]^

Multicomponent non-pharmacological interventions, which combine multiple strategies such as early mobilization, cognitive stimulation, and sleep promotion, have shown promising results in reducing the incidence of POD. The Hospital Elder Life Program (HELP), a multicomponent intervention designed to prevent delirium in hospitalized older adults, has been shown to significantly reduce the incidence and duration of delirium.^[136]^ Implementing multicomponent non-pharmacological interventions tailored to the needs of orthopedic patients may be an effective strategy for preventing POD.

In conclusion, non-pharmacological approaches play a vital role in the prevention of POD in orthopedic patients. These interventions, which include early mobilization, cognitive stimulation, sensory enhancement, sleep promotion, and nutritional support, target the modifiable risk factors for POD and promote cognitive and physical function. Implementing non-pharmacological strategies in conjunction with pharmacological interventions may provide a comprehensive approach to preventing POD in orthopedic patients. Further research is needed to determine the optimal combination and timing of non-pharmacological interventions for preventing POD in this vulnerable population (Table 2).

**Table 2:** Pharmacological and non-pharmacological interventions for the prevention of postoperative delirium in orthopedic patients.

Intervention category	Intervention type	Examples	Reported RR range	Evidence quality	References
Pharmacological	Antipsychotics	Haloperidol, risperidone, olanzapine	0.6–0.8	Moderate	[20,21,114,115]
	Cholinesterase inhibitors	Donepezil, rivastigmine	0.7–0.9	Low	[22,116]
	Melatonin	–	0.7–0.9	Low	[64,117,118]
	Statins	–	0.8–0.9	Low	[119–121]
	Other agents	Dexmedetomidine, gabapentin, ketamine	0.5–0.7	Moderate	[122–124]
Non-pharmacological	Early mobilization	Getting patients out of bed and engaging in physical activity post-surgery	0.4–0.7	High	[1,125–127]
	Cognitive stimulation and orientation	Providing clocks and calendars, engaging in conversations, puzzles, games	0.6–0.8	Moderate	[26,114,128–130]
	Sensory enhancement	Providing hearing aids and eyeglasses, minimizing noise, adequate lighting	0.7–0.9	Low	[26,131,132]
	Sleep promotion	Establishing regular sleep-wake schedule, minimizing nighttime disruptions	0.7–0.9	Moderate	[65,133,134]

Evidence quality based on consistency across studies and methodological rigor.

RR = relative risk.

### 3.4. Treatment strategies for postoperative delirium in orthopedic patients

#### 3.4.1. Pharmacological management

The management of POD in orthopedic patients presents a significant challenge, and pharmacological interventions form a crucial component of the treatment strategy. The primary goals of pharmacological management are to control symptoms, ensure patient safety, and facilitate recovery.^[137]^ The choice of medication depends on the patient’s clinical presentation, underlying medical conditions, and potential drug interactions.^[138]^

Antipsychotics are the most commonly used medications for the treatment of POD. Both typical (e.g., haloperidol) and atypical (e.g., risperidone, olanzapine, quetiapine) antipsychotics have been studied for their efficacy in managing POD.^[139]^ Haloperidol, a first-generation antipsychotic, has been widely used for the treatment of delirium due to its rapid onset of action and favorable side effect profile.^[140]^ However, the use of haloperidol is associated with extrapyramidal side effects and prolonged QTc interval, warranting careful monitoring.^[141]^

Atypical antipsychotics, such as risperidone, olanzapine, and quetiapine, have gained attention as alternatives to haloperidol due to their lower risk of extrapyramidal side effects.^[142]^ These medications have been shown to be effective in reducing the severity and duration of POD, with a more favorable side effect profile compared to haloperidol.^[143]^ However, atypical antipsychotics can still cause side effects, such as sedation and metabolic disturbances, and should be used with caution in elderly patients.^[144]^

Benzodiazepines, such as lorazepam and midazolam, are another class of medications that have been used for the treatment of POD, particularly in patients with severe agitation or alcohol withdrawal.^[145]^ However, the use of benzodiazepines in the elderly population is associated with an increased risk of falls, respiratory depression, and paradoxical agitation.^[146]^ Therefore, benzodiazepines should be used judiciously and only when necessary for the control of severe symptoms.^[147]^

Dexmedetomidine, a selective α-2 adrenergic receptor agonist, has emerged as a promising option for the treatment of POD in critically ill patients.^[148]^ Dexmedetomidine has sedative, anxiolytic, and analgesic properties, without causing significant respiratory depression.^[149]^ Studies have shown that dexmedetomidine can reduce the incidence and duration of POD in patients undergoing cardiac and noncardiac surgery.^[150]^ However, the use of dexmedetomidine may be limited by its hemodynamic side effects, such as bradycardia and hypotension.^[151]^

Cholinesterase inhibitors, such as donepezil and rivastigmine, have been investigated for their potential role in the treatment of POD, based on the cholinergic deficiency hypothesis.^[152]^ These medications have been shown to improve cognitive function and reduce the severity of delirium in patients with Alzheimer’s disease.^[153]^ However, the evidence for their efficacy in the treatment of POD is limited, and further research is needed to establish their role in the management of this condition.^[142]^

Melatonin and melatonin receptor agonists, such as ramelteon, have been studied for their potential role in the treatment of POD, given their involvement in the regulation of sleep-wake cycles.^[154]^ Melatonin has been shown to reduce the incidence and duration of POD in elderly patients undergoing surgery, possibly through its antioxidant and anti-inflammatory properties.^[155]^ However, the optimal dosing and timing of melatonin administration for the treatment of POD remains to be determined.^[156]^

In conclusion, pharmacological management plays a vital role in the treatment of POD in orthopedic patients. Antipsychotics, particularly haloperidol and atypical antipsychotics, are the mainstay of treatment, but their use should be balanced against the risk of side effects. Benzodiazepines should be used with caution, while dexmedetomidine may offer a promising alternative for the management of POD in critically ill patients. Cholinesterase inhibitors and melatonin may have a role in the treatment of POD, but further research is needed to establish their efficacy. The choice of medication should be individualized based on the patient’s clinical presentation, underlying medical conditions, and potential drug interactions, with careful monitoring for side effects and treatment response (Table 3).

**Table 3:** Pharmacological interventions for the management of postoperative delirium in orthopedic patients.

Medication class	Medication examples	Mechanism of action	Reported RR range	Evidence quality	References
Antipsychotics	Typical (e.g., haloperidol); Atypical (e.g., risperidone, olanzapine, quetiapine)	Modulate neurotransmitter imbalances, particularly dopamine and serotonin.	0.6–0.8	Moderate	[139–144]
Benzodiazepines	Lorazepam, midazolam	Used for severe agitation or alcohol withdrawal; associated with increased risk of adverse effects in elderly.	0.7–0.9	Low	[145–147]
Dexmedetomidine	–	Selective α-2 adrenergic receptor agonist with sedative, anxiolytic, and analgesic properties; may reduce the incidence and duration of POD in critically ill patients	0.7–0.9	Low	[148–151]
Cholinesterase inhibitors	Donepezil, rivastigmine	Potential role in the treatment of POD based on the cholinergic deficiency hypothesis; limited evidence for efficacy in POD	0.8–0.9	Low	[142,152,153]
Melatonin and melatonin receptor agonists	Melatonin, ramelteon	Involvement in the regulation of sleep-wake cycles; may reduce the incidence and duration of POD in elderly patients undergoing surgery	0.5–0.7	Moderate	[154–156]

POD = postoperative delirium, RR = relative risk.

#### 3.4.2. Non-pharmacological interventions

The implementation of non-pharmacological interventions is an essential component of the comprehensive management of POD in orthopedic patients. These interventions focus on modifying the hospital environment, optimizing patient care, and promoting cognitive and physical function to alleviate the symptoms of POD and facilitate recovery.^[157]^ Non-pharmacological approaches are particularly valuable as they can be used in conjunction with pharmacological treatments and may help reduce the need for medications that have potential side effects.^[115]^

One of the primary non-pharmacological strategies for managing POD is the promotion of a sleep-conducive environment. Sleep disturbances and circadian rhythm disruptions are common in patients with POD and can exacerbate the condition.^[158]^ Interventions aimed at promoting sleep hygiene, such as maintaining a regular sleep-wake schedule, minimizing nighttime interruptions, and providing a quiet, dark environment, can help improve sleep quality and reduce the severity of POD.^[159]^ The use of ear plugs, eye masks, and white noise machines may also be beneficial in creating a sleep-friendly environment.^[160]^

Cognitive stimulation and reorientation interventions are another important non-pharmacological approach for managing POD. These interventions aim to keep patients mentally engaged and oriented to their surroundings, which can help reduce confusion and disorganized thinking.^[161]^ Examples of cognitive stimulation activities include reminiscence therapy, word games, and discussion of current events.^[130]^ Reorientation strategies, such as providing clear signage, clocks, and calendars in the patient’s room, can help maintain temporal orientation and reduce disorientation.^[136]^

Early mobilization and physical therapy are crucial non-pharmacological interventions for managing POD in orthopedic patients. Immobility and prolonged bed rest can contribute to the development and persistence of POD, as well as other postoperative complications.^[125]^ Encouraging patients to get out of bed, sit in a chair, and engage in light physical activity as soon as possible after surgery can help prevent muscle weakness, improve circulation, and promote cognitive function.^[162]^ Physical therapy exercises, such as range of motion exercises and progressive resistance training, can further enhance functional recovery and reduce the duration of POD.^[163]^

Sensory enhancement interventions, such as providing hearing aids and eyeglasses, can also play a role in managing POD. Sensory impairments can contribute to disorientation and confusion, and ensuring that patients have access to their sensory aids can help them better engage with their environment.^[164]^ Additionally, creating a sensory-friendly environment by minimizing background noise, providing adequate lighting, and offering soothing music can help reduce sensory overstimulation and promote relaxation.^[165]^

Family involvement and social support are important non-pharmacological aspects of POD management. The presence of familiar faces and voices can provide comfort and reassurance to patients experiencing POD.^[166]^ Encouraging family members to visit regularly, assist with reorientation, and engage in calming activities, such as reading or listening to music together, can help reduce anxiety and agitation.^[167]^ Providing education to family members about POD, its symptoms, and how they can support their loved one’s recovery can also be beneficial.^[168]^

Multicomponent non-pharmacological interventions that combine several of the above strategies have shown promise in the management of POD. The Hospital Elder Life Program (HELP), a comprehensive intervention designed to prevent and manage delirium in hospitalized older adults, includes a combination of cognitive stimulation, early mobilization, sleep enhancement, and sensory optimization.^[128]^ Studies have demonstrated that the implementation of HELP can significantly reduce the incidence, duration, and severity of POD in various hospital settings, including orthopedic units.^[169]^

In conclusion, non-pharmacological interventions are a vital component of the multimodal approach to managing POD in orthopedic patients. These interventions, which include sleep promotion, cognitive stimulation, early mobilization, sensory enhancement, and family involvement, target the modifiable risk factors for POD and promote cognitive and physical function. The integration of non-pharmacological strategies with pharmacological treatments may provide a more comprehensive and effective approach to managing POD, while minimizing the potential adverse effects of medications. Further research is needed to determine the optimal combination and timing of non-pharmacological interventions for managing POD in the orthopedic population (Table 4).

**Table 4:** Non-pharmacological interventions for the management of postoperative delirium in orthopedic patients.

Intervention category	Description	Reported effectiveness	Evidence quality	References
Sleep-conducive environment	Maintaining a regular sleep-wake schedule, minimizing nighttime interruptions, providing a quiet, dark environment; use of ear plugs, eye masks, and white noise machines	60–80% improvement	Moderate	[158–160]
Cognitive stimulation and reorientation	Keeping patients mentally engaged and oriented to their surroundings; reminiscence therapy, word games, discussion of current events; providing clear signage, clocks, and calendars	50–70% improvement	Moderate	[130,136,161]
Early mobilization and physical therapy	Encouraging patients to get out of bed, sit in a chair, and engage in light physical activity post-surgery; range of motion exercises and progressive resistance training	70–85% improvement	High	[125,162,163]
Sensory enhancement	Providing hearing aids and eyeglasses; minimizing background noise, providing adequate lighting, offering soothing music	40–60% improvement	Low	[164,165]
Family involvement and social support	Presence of familiar faces and voices; encouraging regular visits, assisting with reorientation, engaging in calming activities; providing education about POD and how to support recovery	50–70% improvement	Low	[166–168]
Multicomponent interventions (e.g., Hospital Elder Life Program)	Combining multiple non-pharmacological strategies (e.g., cognitive stimulation, early mobilization, sleep enhancement, sensory optimization) to manage POD	70–90% improvement	High	[128,169]

POD = postoperative delirium.

## 4. Conclusion and perspectives

This integrative review reveals critical knowledge gaps that warrant prioritized research attention. Specific research priorities include – long-term cognitive outcomes: limited evidence exists on POD’s impact beyond hospital discharge, particularly cognitive trajectory and functional recovery in orthopedic patients^[170,171]^; personalized risk stratification: current risk models lack validation across diverse orthopedic populations and surgical types, hindering individualized prevention strategies^[172]^; biomarker development: inflammatory and neurological biomarkers for early POD detection remain understudied in orthopedic contexts^[173]^; and implementation science: evidence-practice gaps persist regarding multimodal intervention adoption in real-world orthopedic units.^[174]^

Novel insights from this review include: POD in orthopedic surgery exhibits distinct risk profiles compared to general surgery, with mechanical factors (immobilization, pain) playing larger roles than traditionally recognized. The interaction between surgical trauma-induced neuroinflammation and age-related brain vulnerability appears more pronounced in orthopedic procedures, suggesting procedure-specific prevention protocols may be more effective than generic approaches.

Future research should prioritize: prospective cohort studies examining 6 to 12 month cognitive outcomes post-orthopedic surgery; development and validation of orthopedic surgery-specific POD prediction models incorporating biomarkers; pragmatic trials testing implementation strategies for multimodal prevention programs in diverse healthcare settings; and mechanistic studies elucidating the neuroinflammation-immobilization interaction in POD pathogenesis.

Clinical implications: healthcare systems should develop orthopedic surgery-specific POD protocols emphasizing early mobilization, pain optimization, and sleep preservation. Investment in staff training for non-pharmacological interventions and standardized delirium assessment tools specific to postsurgical orthopedic patients is essential for translating this evidence into improved patient outcomes.

## Acknowledgments

We would like to take this opportunity to express our sincere gratitude to West China Hospital, Sichuan University, for their strong support of this research.

## Author contributions

**Conceptualization**: PengCheng Li.

**Data curation**: PengCheng Li.

**Funding acquisition**: Jian Li.

**Funding acquisition**: Jian Li.

**Supervision**: PengCheng Li.

**Resources**: Jian Li.

**Writing – original draft**: Lian Huang.

**Writing – review & editing**: PengCheng Li.
